# Impairments in Cognitive Control Using a Reverse Visually Guided Reaching Task Following Stroke

**DOI:** 10.1177/15459683221100510

**Published:** 2022-05-16

**Authors:** Catherine R. Lowrey, Sean P. Dukelow, Stephen D. Bagg, Benjamin Ritsma, Stephen H. Scott

**Affiliations:** 1 Centre for Neuroscience Studies, 4257 Queen’s University, Kingston, ON, Canada; 2 Hotchkiss Brain Institute, 2129 University of Calgary, Calgary, AB, Canada; 3 Department of Physical Medicine and Rehabilitation, 4257 Queen’s University, Kingston, ON, Canada; 4 School of Medicine, 4257 Queen’s University, Kingston, ON, Canada; 5 Department of Biomedical and Molecular Sciences, 4257 Queen’s University, Kingston, ON, Canada; 6 Department of Medicine, 4257 Queen’s University, Kingston, ON, Canada

**Keywords:** stroke, precision medicine, exoskeleton device, robotics, neurosciences

## Abstract

**Background:**

Cognitive and motor function must work together quickly and seamlessly to allow us to interact with a complex world, but their integration is difficult to assess directly. Interactive technology provides opportunities to assess motor actions requiring cognitive control.

**Objective:**

To adapt a reverse reaching task to an interactive robotic platform to quantify impairments in cognitive-motor integration following stroke.

**Methods:**

Participants with subacute stroke (N=59) performed two tasks using the Kinarm: Reverse Visually Guided Reaching (RVGR) and Visually Guided Reaching (VGR). Tasks required subjects move a cursor “quickly and accurately” to virtual targets. In RVGR, cursor motion was reversed compared to finger motion (i.e., hand moves left, cursor moves right). Task parameters and Task Scores were calculated based on models developed from healthy controls, and accounted for the influence of age, sex, and handedness.

**Results:**

Many stroke participants (86%) were impaired in RVGR with their affected arm (Task Score > 95% of controls). The most common impairment was increased movement time. Seventy-three percent were also impaired with their less affected arm. The most common impairment was larger initial direction angles of reach. Impairments in RVGR improved over time, but 71% of participants tested longitudinally were still impaired with the affected arm ∼6 months post-stroke. Importantly, although 57% were impaired with the less affected arm at 6 months, these individuals were not impaired in VGR.

**Conclusions:**

Individuals with stroke were impaired in a reverse reaching task but many did not show similar impairments in a standard reaching task, highlighting selective impairment in cognitive-motor integration.

## Introduction

A broad range of brain functions can be impacted following stroke since lesions can vary dramatically in size and location. Motor impairments are common and have been the predominant focus for assessment and rehabilitation. However, cognitive impairments are also common such as attention and memory problems, loss of inhibition and executive function.^1-3^ Stroke may also interfere with coupling between cognitive and motor functions which is important for daily activities that are time sensitive such as driving a vehicle or walking down a crowded street. Thus, cognitive and motor functions must interact quickly and seamlessly to allow us to move within a complex world, but this interaction cannot be adequately quantified through traditional clinical tools.

Interactive computer and robotic platforms provide can assess cognitive-motor interactions^4-8^ as they allow the development of tasks to quantify speed and accuracy of motor actions that require cognitive processes. We recently developed a task where individuals were required to hit certain “target” objects but avoid other “distractor” objects.^4^ Many participants with stroke hit fewer objects overall, similar to a simpler version of the task^9^ that included targets only. However, this task also identified 15% of individuals with stroke that hit a higher ratio of distractor objects than healthy controls, in some cases, hitting a similar number of targets as distractors, suggesting a selective impairment in inhibitory control.

Another paradigm to study cognitive control of motor actions is the reverse reaching task. This task was developed on a computer touch screen where cursor motion was reversed (e.g., finger moves right, cursor moves left)^5^ and is akin to an anti-saccade task, where participants are instructed to look in the opposite direction when a spatial target is displayed.^10^ Like the anti-saccade task, reverse reaching requires cognitive control to inhibit arm movement towards the target, and instead, generate a movement in the opposite direction.^11-15^ As well, reverse reaching requires continual cognitive control to support online control of limb motion to guide the cursor toward the target. Whereas healthy individuals are typically able to learn this task within a few trials, task performance is worse in individuals with increasing cognitive impairment.^5,16,17^ Interestingly, the task is able to identify impairments in asymptomatic individuals with a history of concussion,^18^ and even pre-symptomatic individuals with a family history of Alzheimer’s Disease,^19^ indicating the sensitivity of the task for identifying even slight impairments in cognitive control. This task also identified performance differences from control in a group of 9 participants with chronic stroke (3–19 years post-stroke) with right fronto-parietal lesions.^20^

The ability to complete a “reverse reach” may not appear relevant for performing everyday activities, but the underlying skill to generate complex motor actions is pertinent to many daily functions. For example, using indirect visual feedback from a mirror for grooming or dressing, or using a rearview mirror to reverse a vehicle. Similarly, reverse reaching involves the skill of attaining a behavioral goal that is complexly related to arm/hand motion. Tool use, by definition, involves translating body movements into a behavioral goal using an object, such as using a pair of scissors or rotating a steering wheel to drive a vehicle. Success in reverse reaching requires hand motion to be converted into the motion of an object (the cursor) towards a target, and thus is a form of tool use. Therefore, reverse reaching provides an opportunity to objectively quantify the underlying skills necessary for complex tasks of daily living, many of which are impacted following stroke.

Our objective was to adapt the reverse reaching task to an interactive robotic platform and quantify impairments associated with sub-acute stroke. We compared performance of participants with stroke to a large database of healthy controls to identify impairments in performance. We hypothesized that since reverse reaching requires a broad range of abilities, beyond simply the motor skill to reach to a target, that participants with stroke would show greater impairments on this task compared to a simple reaching task.

## Methods

### Participants

A cohort of control participants were recruited from Kingston, Ontario, Canada and were included if they were ≥18 years of age and could understand task instructions. They were excluded if they had neurologic or musculoskeletal diagnoses affecting the upper limbs. These participants form a large database of robotic task data. Participants with stroke were recruited from St Mary’s of the Lake and Providence Care Hospitals in Kingston and were included if they had a diagnosis of stroke and could understand task instructions. Participants were excluded if they had significant medical comorbidities (e.g., angina or active cardiac disease), a previous stroke, or other neurologic or upper limb musculoskeletal diagnoses. Participants provided informed consent. This study was approved by Queen’s University Health Sciences and Affiliated Teaching Hospitals Research Ethics Board (#ANAT042-05).

### Clinical Examinations

A physical therapist administered clinical evaluations of stroke participants: Modified Edinburgh Handedness Inventory, Montreal Cognitive Assessment (MoCA; scored out of 30, scores <26 indicate mild cognitive impairment),^21^ Behavioral Inattention Test (BIT; conventional subsection; scored out of 146 and <130 is indicative of visual neglect),^22^ Chedoke–McMaster Assessment of the arm and hand (CMSA; 7=highest recovery stage, 1 = lowest recovery stage),^23^ and Functional Independence Measure (FIM^TM^; scores from 18–126; 18 = complete dependence, 126 = complete independence^24^; see Supplementary Table 1). Clinical tests determined the most affected side of the body, which is referred to as “affected side” throughout. Some individuals with stroke also experience impairments in the other arm,^25-30^ and so is referred to as the “less affected” side.

### Robotic Set-Up

**Figure 1.**
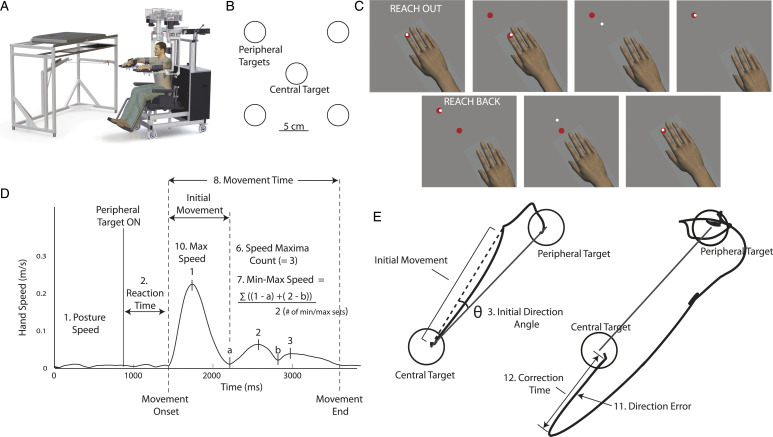
(A) Schematic illustration of the Kinarm exoskeleton robotic platform. (B) Placement of targets in VGR and RVGR. (C) Visualization of RVGR task. Top pictures show reach out phase and bottom pictures show reach back. Note that white hand feedback dot moves in opposite direction as the hand. Hand is shown in the visualization but the arms and hands were occluded from participants' view for the duration of the task. (D) Schematic diagram of hand speed. (E) Trace of hand paths for two reaches outs from the central target to the peripheral target. On the left, participant reached straight to the target (i.e., with no direction error). Solid black line is a straight line between two target centers. Dashed line indicates initial movement. The example reach trajectory on the right shows a direction error when the participant begins the reach by moving in the direction away from the peripheral target. The correction time is calculated from the start of the direction error to the time when the subject started to move the cursor back towards the peripheral target.

Participants performed tasks in the Kinarm exoskeleton lab (Kinarm, Kingston, Ontario; https://kinarm.com/kinarm-products/kinarm-exoskeleton-lab/). Set-up details have been previously described.^25^ Briefly, participants sat in a chair that could provide truncal support, with arms supported in exoskeleton robots. Seat height was adjusted so shoulder was abducted ∼85°. Shoulder and elbow joints were aligned with robot linkages (Figure 1A). Arms rested in plastic troughs, adjusted to support the upper arm and forearm/hand and allow free movement in the horizontal plane. The robot was calibrated for each participant. During testing, arms and hands were occluded from view. A virtual reality system projected visual targets and a visual representation of the index fingertip location on the screen, and aligned in the same horizontal plane as the arm.

### Experimental Task

Reverse Visually Guided Reaching task (RVGR): participants reached to a central target (red circle, 1 cm radius; Figure 1B) using a cursor (white circle, 0.5 cm radius) that paralleled the motion of the index finger. Once the cursor reached the central target, the relationship between cursor and hand motion flipped, so that the cursor moved 180° opposite to motion of the finger (Figure 1C). After a random time (750–1250 ms) one of four peripheral targets appeared. Peripheral targets were spaced 90° apart, 10 cm from center (Figure 1B). Participants were required to reach the peripheral target within 6 seconds. Once the cursor reached the target, or 6 seconds elapsed, the central target reappeared and participants reached back to center. Targets were presented in 6 blocks of 4. Each of the 4 targets was presented randomly within a block. Participants received verbal instructions before the task: “In this task you will have a white light that represents your fingertip and a red light. For the first trial, put the white light in the red light. The white light will then move in the opposite direction of what you expect. Please try to put the white light in the red light quickly and accurately.”

Participants also completed a Visually Guided Reaching task (VGR^25^), which was identical to RVGR, but cursor motion always paralleled hand motion. VGR was performed first, followed by RVGR. The order of the arms (right or left) was randomized.

### Data Analysis

Robot position and velocity were recorded at 1000 Hz and using Dexterit-E software (versions 3.4–3.8, Kinarm, Kingston, ON, Canada). Hand position, speed, and acceleration were calculated and analyzed using Dexterit-E software (version 3.8, Kinarm, Kingston, ON, Canada). Signals were filtered (sixth-order double-pass Butterworth low-pass filter) with a 10 Hz cutoff frequency.

### Task Performance Measures

Task parameters were calculated to quantify performance (Figure 1D and E). Parameters were developed for VGR^25^ (see Supplemental Material for parameter descriptions). Two additional parameters were developed for RVGR: Direction Error and Correction Time. Direction Errors were the number of times subjects initially moved the cursor away from the peripheral target. If a Direction Error occurred, Correction Time was the amount of time before the subject started to move the cursor back towards the peripheral target.

Performance of stroke participants was compared to normalized models of behavior calculated from a large database of healthy controls (∼250–500 participants spanning 18–93 years of age, ∼50% female; Table 1). This process has been outlined previously^31,32^ and the detailed procedure can be found in Kinarm Standard Test Summary (https://kinarm.com/download/kst-summary-analysis-version-3-9). Briefly, control data were transformed to a standard, normal distribution using Box-Cox transformations^33^ and fit using weighted linear regression to account for age, sex and handedness. Parameter values were thus transformed to a standard “Z-score,” reflecting performance in units of standard deviations. A Z-score ≤ −3.29 or ≥ +3.29 (1 in 1000 or >99.9%) was classified as an outlier and removed from the control dataset. Data normalization and outlier removal was then repeated. Renormalization without these outliers necessarily reduces the breadth of the remaining distribution and can result in more data points to now become outliers. Therefore, we repeated this normalization process at most three times to limit the repeated loss of datasets.

**Table 1.** Participant Characteristics.

Control participants							
	VGR (N = 514)				RVGR (N = 288)		
Age (years)	18–93				18–84		
Sex (male/female)	220/294 subjects				127/161 subjects		
Stroke participants (N = 59)							
age (years)	26–94						
sex (Male/female)	32/27 subjects						
handedness (Left/right)	9/50 subjects						
time Since stroke	27 (8–73) days						
affected Arm (L/R/Both)	29/28/2						
FIM- cognitive sub-score (/35)	30.8 (21–35)						
FIM- motor sub-score (/91)	69.5 (41–91)						
FIM- total score (/126)	100.3 (62–124)						
MoCA	24.2 (10–30)						
BIT	141.7 (117–146)						
Lesion location (number of subjects)^a^	C	SC	C + SC	Cb		Br	UK
	19	26	7	3		4	0
Ischemic/hemorrhagic/unknown	43/13/2 subjects						
BIT <130	2 subjects						
visual Field deficit	2 subjects						
CMSA-arm subscore	[ 7 6 5 4 3 2 1]^b^						
Affected arm [# of subjects]	[13 4 3 2 14 13 6]						
Unaffected arm	[30 19 5 0 1 0 0]						
Affected hand	[ 9 13 10 1 8 10 4]						
Unaffected hand	[35 18 2 0 0 0 0]						

FIM: Functional Independence Measure, MoCA: Montreal Cognitive Assessment, BIT: Behavioral Inattention Test; CMSA: Chedoke–McMaster Stroke Assessment.

^a^C: Cortical; SC: Subcortical; C + SC: Cortical and Subcortical; Cb: Cerebellum; Br: Brainstem; Uk: Unknown.

^b^Scoring for the CMSA: 7 – highest recovery 1 – poorest recovery.

Task Scores were calculated using parameter scores to provide a measure of task performance. Parameter Z-scores were transformed (0 = best performance, higher values = poorer performance). Root-sum-squares of these standardized scores was performed, then transformed to normal using Box-Cox equations and further transformed to a Task Score (where 0 = best performance, higher values = poorer performance). If Task Score was greater than 3.09 (Z-score = 3.29 or 99.9%), that participant was removed as an outlier.

Critically, the same parameter models developed from controls are then used to convert raw parameter scores of participants with stroke to Z-scores. By calculating a Z-score for participants with stroke we can identify if they are impaired compared to the expected performance of individuals of a similar age, sex and handedness, as these are accounted for in the models of healthy performance. Performance for individual participants on a given parameter was identified as impaired if it fell outside of 95% of controls; that is, a Z-score > 1.65 (see Supplementary Table 2). Task Scores were also calculated using the model developed from controls and were converted to Z-scores for all calculations and analyses (Z-Task Scores). Task Scores were identified as impaired if they were greater than 1.96 (>95% of healthy controls). Z-Task Scores were identified as impaired if they were >1.65.

### Statistical Analyses

Participants were classified as Right- or Left-Affected (RA or LA) if the right or left side of their body was identified as most affected, respectively. Z-Task Scores for these groups were not normally distributed (Lilliefors test, p > 0.05) and were compared using Wilcoxon rank sum comparisons.

Data were collected initially at T1, ∼1 month following stroke. For a subset of participants (N=14) data were collected at T2, ∼3 months and T3, ∼6 months post-stroke. Z-Task Scores at T1–T3 were not normally distributed (Lilliefors test, p>0.05) and were compared using Wilcoxon’s signed rank test (T1 vs T2, T2 vs T3, T1 vs T3) for each arm (6 comparisons per task). Calculated p-values were Bonferroni-adjusted (p-values*6).

A previous study identified significant change and learning effects based on repeat testing on neurologically healthy participants for RVGR and VGR.^34^ Significant change for Z-Task Score is 1.05 (VGR) and 1.34 (RVGR) (dominant arm) and 2.19 (VGR) and 1.79 (RVGR) (non-dominant arm).^34^ Learning effects were also taken into account when determining changes between T1 and T2 for RVGR: −0.78 for the dominant arm and −0.67 for the non-dominant arm.

Intra-class correlation coefficients (ICC; r-values) were used to evaluate inter-rater reliability of RVGR, for 42 controls and 10 participants with stroke who were set up in the robot by two different Kinarm operators, no more than 1 week apart (majority were completed on the same day). ICC estimates and their 95% confidence intervals were calculated using MATLAB based on a single-rating (k = 1), consistency, 2-way mixed-effects model. The consistency model was chosen to take into account learning effects which have been previously observed for the RVGR task.^34^

Correlations between Z-Task Scores and 7 clinical tasks were performed using Spearman’s rank correlation (Bonferroni correction: 7 comparisons per arm; p-values *7).

## Results

### Participant Characteristics

Data were collected from 59 participants with stroke and compared to the database of healthy controls; (N = 514 (VGR), N = 288 (RVGR); Table 1). Full exams were collected for all participants with stroke but one, for whom RVGR could not be completed on the affected arm. Data were collected within the subacute phase, ∼1 month following stroke (8–73 days; T1).

### Exemplar Participant Performance

**Figure 2.**
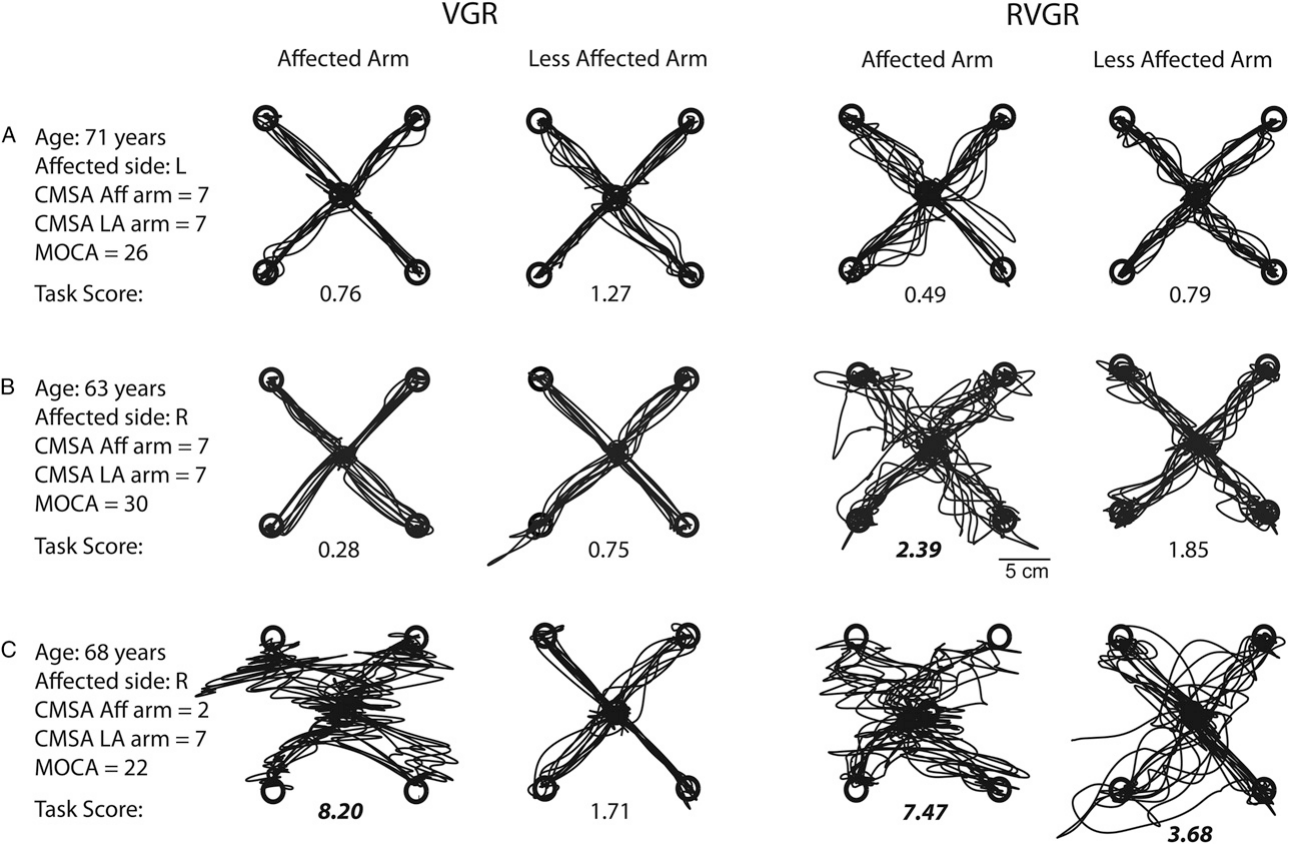
A-C Exemplar participant hand traces for VGR (two left traces) and RVGR (two right traces). Task Score displayed below hand traces with bold denoting scores >1.96 (identified as impaired).

Participants were generally more variable when reaching to targets in RVGR and less successful at reaching targets. Figure 2 shows common patterns of impairment across tasks and arms. Panel A shows a 71-year-old participant with stroke with modest functional challenges (CMSA aff arm = 7, CMSA less aff arm = 7, FIM = 116, BIT = 145, MoCA = 28). Hand paths for both tasks are relatively straight and Task Scores (TS) were <1.96, indicating that performance on either task was not impaired compared to controls. Panel B shows hand traces from a 63-year-old participant with stroke (CMSA aff arm = 7, CMSA less aff arm = 7, FIM = 124, BIT = 145, MoCA = 30). Highly variable paths were seen in RVGR, particularly for the affected arm and performance was impaired compared to controls (TS_(affected)_ = 2.39). In contrast, Task Scores indicate no impairments in RVGR with the less affected arm and no impairments with either arm in VGR. Panel C shows hand traces for a 68-year-old participant with stroke (CMSA aff arm = 2, CMSA less aff arm = 7, FIM = 88, BIT = 140, MoCA = 22). Hand paths were variable in RVGR and performance on the task was impaired in both arms compared to controls (TS_(affected)_ = 7.47, TS_(less affected)_ = 3.68). Performance of the affected arm on VGR was impaired compared to controls (TS_(affected)_ = 8.20) but performance of the less affected arm was not impaired (TS_(less affected)_ = 1.71).

### Stroke Group Performance – RVGR

**Figure 3.**
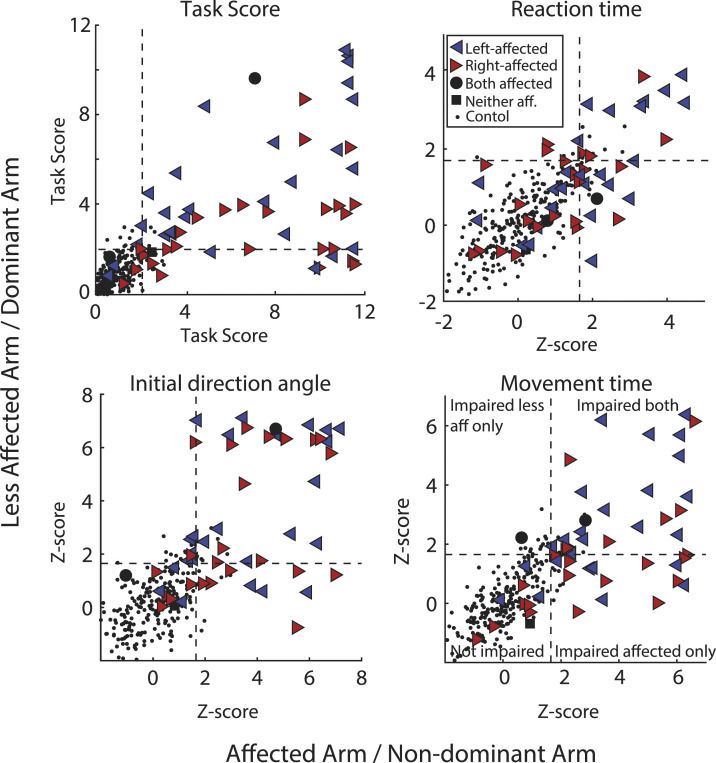
Task Scores and Z-scores for selected task parameters for RVGR. Right-facing red triangles represent right affected (RA) participants where the most affected arm is the right, left-facing blue triangles represent left affected participants (LA). Small black dots are control participants. Dashed lines represent the cutoff for 95% of healthy controls (i.e., Task Score = 1.96; Z-score = 1.65). Scores are plotted for the affected arm (or non-dominant arm for controls) compared to the less affected arm (dominant arm).

The majority of participants (67.8%) were impaired on RVGR with both arms (Task Scores, Figure 3; Table 2). Movement Time flagged the most impairments with the affected arm (78.8%); meaning almost 80% of participants with stroke moved with longer movement times than 95% of controls. Initial Direction Angle flagged the most impairments with the less affected arm (64.4%), meaning 64% of participants with stroke moved with a greater deviation from a straight line from start target to end target. Of note, although only a few participants with stroke (3.4%) made significantly more Direction Errors with their affected arm, 44.3% showed impairments in Correction Time. More participants showed impairments in Direction Errors with their less affected arm (44%) than with their affected arm (3.4%). See Supplementary Table 4 for percentage of participant impairments in all RVGR and VGR parameters.

**Table 2.** Results.

	Stroke - % of participants impaired		ICC consistency		
	(N = 59)		(N = 42 Control, N = 10 Stroke)		
Parameter	affected (%)	Less affected (%)	r	LB	UB
Posture speed	41.5	32.8	0.41	0.23	0.56
Reaction time	44.2	28.8	0.56	0.42	0.68
Initial direction angle	67.3	64.4	0.63	0.5	0.73
Initial distance ratio	5.8	3.7	0.73	0.63	0.81
Initial speed ratio	42.3	32.2	0.72	0.62	0.8
Speed maxima count	73.1	52.5	0.72	0.61	0.8
Min-max speed	50	38.9	0.82	0.75	0.87
Movement time	78.8	50.9	0.75	0.65	0.82
Path length ratio	69.2	57.6	0.79	0.71	0.85
Max speed	21.2	18.6	0.83	0.75	0.88
Direction errors	3.44	20.3	0.9	0.86	0.93
Correction time	44.2	54.2	0.85	0.79	0.9
No initial stabilization	71.2	42.4	0.87	0.81	0.91
No reaction time	71.2	40.7	0.88	0.83	0.92
No end movement	71.2	42.4	0.93	0.89	0.95
End target not reached	75.9	50.8	0.92	0.89	0.95
Task score	86.2	72.9	0.92	0.89	0.95

### RVGR – Right vs Left Affected Participants With Stroke

Being right- (RA) or left-affected (LA) influenced the performance with the less affected arm on the RVGR task. RVGR Z-Task Scores (Z-TS) with the less affected arm were significantly higher (worse) for LA participants than RA participants (Z-TS_LA(less affected)_ = 4.53, Z-TS_RA(less affected)_ = 2.61, p = 0.006). These differences were not seen for the affected arm (Z-TS_LA(affected)_ = 6.36, Z-TS_RA(affected)_ = 6.49, p = 0.45). There were no significant differences in VGR between RA and LA participants for either the affected or less affected arm (Z-TS_LA(affected)_ = 5.34, Z-TS_RA(affected)_ = 6.47, p = 0.17; Z-TS_LA(less affected)_ = 1.97, Z-TS_RA(less affected)_ = 2.09, p = 0.42).

### Stroke Group Performance – VGR vs RVGR

**Figure 4.**
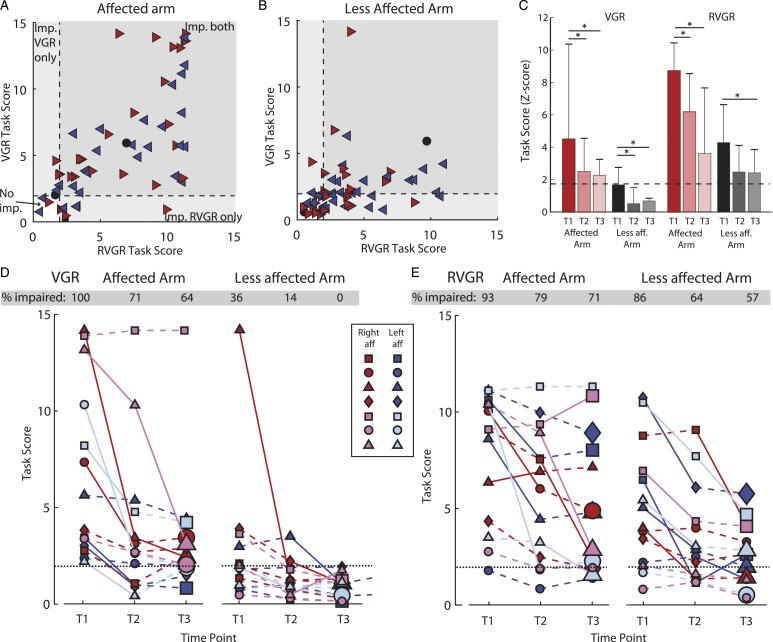
(A) Task Scores for affected arm for RVGR compared to VGR task. Dashed lines represent the cutoff for 95% of healthy controls (i.e., Task score = 1.96). Right-facing red triangles represent right affected (RA) participants where the most affected arm is the right, left-facing blue triangles represent left affected participants (LA). Black circles represent participants where both arms were equally affected and black squares represent participants where neither arm was affected. Small black dots are control participants (B) Same as (A) but for the less affected arm. (C) Group medians for each task for the affected and less affected arms. Error bars represent the 75^th^ percentile. (D) Task scores for the VGR task plotted longitudinally for a subset of participants with stroke (N = 14, 7 RA, 7 LA). Each icon and color combination represents a unique participant. Solid lines between consecutive time points indicate a significant change. Dashed lines between consecutive time points represent a non-significant change. Large icons at T3 represent a significant change from T1 to T3. E) Same as D) but for RVGR.

A large portion (74%) of participants with stroke were impaired on both tasks with their affected arm (Figure 4A, Tables 2 and 45.8% were impaired on both tasks with their less affected arm (Figure 4B, Table 2). Of interest, 27.1% were impaired on RVGR but not VGR with their less affected arm.

### Stroke Group Performance – Longitudinal

Data were collected for a subset of participants (N = 14) at T2 (∼3 months, 72-114 days post stroke) and T3 (∼6 months, 127–329 days post stroke). As a group, performance with the affected arm significantly improved at T2 for both tasks (Median Z-Task Scores: Z-TS_VGR(T1)_ = 3.65, Z-TS_VGR(T2)_ = 2.46, p_adj_ = 0.004; Z-TS_RVGR(T1)_ = 9.06, Z-TS_RVGR(T2)_ = 6.36 p_adj_ = 0.036). There were no significant differences between group scores at T2 and T3 for either task. Performance with the less affected arm significantly improved at T2 and T3 for the VGR task (Z-TS_VGR(T1)_ = 1.51, Z-TS_VGR(T2)_ = 0.35, p_adj_ = 0.048, Z-TS_VGR(T3)_ = 0.53, p_adj_ = 0.007). Performance with the less affected arm in RVGR did not significantly improve at T2, but did significantly improve at T3 (Z-TS_RVGR(T1)_ = 4.35, Z-TS_RVGR(T2)_ = 2.43, p_adj_ = 0.102, Z-TS_RVGR(T3)_ = 2.38, p_adj_ = 0.002).

In general, individual participant recovery was greater in VGR compared to RVGR. Significant improvement (lower Task Score = improved task performance) was calculated based on previous work^34^ and took into account any expected learning effects between repeated task session (see methods). With their affected arm, 8 participants significantly improved Task Score in VGR from T1 to T2 (Figure 4D); whereas in RVGR only 4 participants improved and 1 participant was significantly worse (Figure 4E). Despite these improvements, 64% and 71% of participants were still impaired at T3 compared to controls (Task Score >1.96) in VGR and RVGR, respectively.

With the less affected arm, for VGR, 2 participants showed significant improvement from T1 to T2 and an additional 1 showed improvements from T2 to T3. In RVGR, 7 participants significantly improved from T1 to T2, and an additional 2 significantly improved from T2 to T3. Of note, at the T3 time point, 0% of participants were impaired in VGR with the less affected arm, whereas 57% of participants were still impaired in RVGR.

### Inter-Rater Reliability

In order to assess the inter-rater reliability of RVGR, a subset of participants (stroke (n=10); control (n=42)) completed the task twice, less than a week apart, set up by a different operator each time. Most parameters (10/16) had ICC values >0.75 (good to excellent; Table 2).^35^ The rest of the parameters had moderate ICC values (0.50–0.74) and one parameter, Posture Speed, had an ICC value of 0.41 (poor). Task Scores had an ICC value of 0.92 (excellent).

### Clinical Correlations

Z-Task Scores from both tasks were significantly negatively correlated with CMSAarm scores for the affected arm (RVGR/CMSAarm: r = −0.65, VGR/CMSAarm: r = −0.82). Affected arm performance on both tasks was also negatively correlated with FIMmotor (RVGR/FIMmotor: r = −0.44, VGR/FIMmotor: r = −0.69) and FIMtotal test scores (RVGR/FIMtotal: r = −0.42, VGR/FIMtotal: r = −0.64). Of note, less affected arm performance on the VGR task was significantly negatively correlated with MoCA scores (VGR/MoCA: r = −0.39) indicating that poorer performance on VGR was associated with poorer performance on MoCA, but less affected arm performance on RVGR was not significantly correlated with MoCA (See Supplemental Material for all correlations).

## Discussion

We adapted a reversed visually guided reaching (RVGR) task for an interactive robotic system and quantified impairments following stroke. Eighty-six percent of stroke participants were impaired on RVGR with their most affected arm as revealed by Task Score. Of note, over 70% were also impaired on the task with their less affected arm. In comparison, only 50% of participants were impaired in a standard reaching task (VGR) with the less affected arm.

An inherent challenge when quantifying impairments compared to healthy individuals is the need to collect large amounts of data. A large sample of healthy individuals that spans most ages, both sexes and handedness, is needed for reliable estimates of performance mean, standard deviation and skew. Typically, this requires hundreds of healthy individuals. However, the benefit of using this type of statistical model is that we can identify patient-specific impairments. For example, we can assess performance of a 75-year-old male patient, based on what is expected for a typical 75-year-old male. A further advantage is that once a normative model is established, it eliminates the need to collect age- and sex-matched controls for each study, as these factors are already considered in the models.

A high prevalence of impairments in reverse reaching is observed, not only after stroke, as shown in current and previous work,^20,36^ but also in other neurological injuries and disorders such as transient ischemic attack,^32^ Alzheimer’s Disease,^5^ amyotrophic lateral sclerosis (ALS),^37^ multiple sclerosis,^38^ epilepsy,^39^ and concussion.^18^ Impairments have also been shown in primarily non-neurological patient groups such as chronic kidney disease patients receiving dialysis^40^ and patients following critical care.^41^ The breadth of patient groups that display impaired performance on variants of this task is likely related to the highly distributed brain network required to perform this complex motor action. This task requires neural circuits involved in goal-directed voluntary movement, such as fronto-parietal circuits,^42^ along with those of the basal ganglia^43^ and cerebellum.^44^ As well, similar to the anti-saccade task, successful task completion requires frontal cortical and supporting neural circuits to provide inhibitory control to suppress the automatic reach towards the visual target, as well as generate a cognitive rule to initiate and control hand movement in the direction opposite to the target.^12,15^ Injury or pathology in any of these networks may lead to impairments in task performance, thus making this a useful task to broadly assess sensorimotor and cognitive processes and their integration.

At the same time, impairments in RVGR could be difficult to interpret as they may stem from disruptions in many different brain regions. It may be possible to gain insight into the nature of the impairments by exploring specific task parameters. For example, Direction Errors, where the individual initially moved the hand towards rather than away from the target, may indicate deficits in inhibitory control^13,45^ Further, assessment of both affected and less affected arms in RVGR revealed that while in some patients, impairments were predominantly in the affected arm, in other cases both arms were impaired, suggesting a general impairment in cognitive control. Another strategy we used to tease apart cognitive vs motor function was to contrast performance in RVGR with the simpler VGR task that requires minimal cognitive control. Some individuals were identified as equally impaired in RVGR and VGR, suggesting that impairments are predominantly motor-related. However, some individuals were selectively impaired in only the RVGR task, and in many cases, the level of impairment was much higher for the RVGR task, highlighting impairments in cognitive-motor control.

A previous study demonstrated impairments in a computer-based reverse reaching task in 9 individuals with chronic stroke with right fronto-parietal lesions.^20^ This prior investigation only tested the ipsilesional arm (i.e., less affected, right arm; personal communication from the authors). Our longitudinal data support and extend these previous findings as 70% of participants tested at ∼6 months following stroke still showed impairments in RVGR with the contralesional (most affected) arm. Further, we extend the previous study’s findings to show impairments following lesions in the left hemisphere. Some individuals tested longitudinally displayed improvements in affected arm VGR without similar improvements in RVGR. Impairments tended to improve more for the less affected arm compared to the affected arm which may be related to the greater use of the less affected arm in daily activities and/or lack of rehabilitation techniques to target cognitive-motor integration.

### Clinical Correlations

Performance on RVGR significantly correlated with some clinical tests but not others. Worse performance on RVGR was correlated with worse performance on FIM and CMSA, but was not correlated with the cognitive portion of FIM, MoCA or BIT. This is somewhat surprising since the latter three tests measure cognitive performance, and it seems intuitive that these would be related to increased cognitive demand of RVGR. However, performance on these tests may differ substantially from RVGR as they have no time constraint and take into account multiple domains of cognition (e.g., memory, language, recall, comprehension, expression, etc.). In contrast, RVGR assesses spatiotemporal features of movement, and thus, emphasizes the interaction of cognitive processes with motor action.

Therefore, it is important to note that while we use the general term “cognitive” to describe RVGR, we recognize that this task captures specific aspects of cognition. Successful completion of the task requires cue-suppression to inhibit the automatic response to reach to a spatial goal. Further, participants must create an arbitrary rule on how to move to the target based on moving the hand in the opposite direction. This is a relatively complex problem, likely involving short-term memory and the development of a novel rule to override the automatic motor system including corrective movements from sensory feedback. In future, we will compare the relationship between RVGR and more standard cognitive tasks (which are being generated for the robotic platform) such as Trail Making, Go-No Go, Stop Signal, and Symbol Digit Modality tests. Inclusion of tests of other fronto-parietal cognitive executive functions will allow more detailed exploration of correlations with RVGR performance.

### Future Directions

Here, we used an interactive robotic system to perform reverse reaching; however, it is important to note that a highly specialized system is not necessary to perform the task. It was originally developed for use on computer/tablet-based systems.^5,17,18,20^ However, there are advantages and disadvantages inherent in using simpler technologies or interactive robotic systems. Computer/tablet-based systems are cheaper, easier to set-up, and also portable allowing their use at bed-side in hospital or home, or at the sidelines in an athletic setting (i.e., to quickly assess a concussed player). However, a robotic platform with augmented reality provides an immersive environment. Participants’ arms are occluded from view, further challenging cognitive, sensory, and motor interactions. The support of exoskeletons allows individuals with severe arm impairments to be assessed, as well as individuals with severe hand impairments as they are not required to point or grasp a touchscreen. The value of interactive robotic systems is that they permit a broad range of other tasks^32,40^ (i.e., robots apply forces or motion to the limb to assess proprioception or corrective responses^4,46^), and provide a broad-based assessment in a well-controlled environment. Future work will likely find that these technologies are complementary, with cheaper, more portable platforms supporting assessments in a variety of healthcare and community environments, and interactive robotics supporting broader, controlled assessment in research and tertiary care hospitals.

## Conclusions

RVGR quantified impairments in motor and cognitive integration. Performance of the less affected arm in RVGR showed greater impairment in the subacute phase than a simple reaching task. These impairments do not recover as well as more simple motor tasks since no impairments were found after 6 months for VGR with the less affected arm but 50% of participants were still impaired with the less affected arm in RVGR.

## Supplemental Material

Supplemental material - Impairments in Cognitive Control Using a Reverse Visually Guided Reaching Task Following Stroke Click here for additional data file. Supplementary material for Impairments in Cognitive Control Using a Reverse Visually Guided Reaching Task Following Stroke by Catherine R. Lowrey, Sean P. Dukelow, Stephen D. Bagg, Benjamin Ritsma, and Stephen H. Scott in Neurorehabilitation and Neural Repair

## Appendix

Abbreviations: RVGR: Reverse Visually Guided Reaching; VGR: Visually Guided Reaching.
