# Supplementary material for: Impairments in Cognitive Control Using a Reverse Visually Guided Reaching Task Following Stroke
Source: Neurorehabil Neural Repair. 2022 May 16;36(7):449–60. doi: 10.1177/15459683221100510 (PMC9198399; doi:10.1177/15459683221100510)
Supplement: Supplemental material - Impairments in Cognitive Control Using a Reverse Visually Guided Reaching Task Following Stroke [file sj-pdf-1-nnr-10.1177_15459683221100510.pdf]

SUPPLEMENTAL MATERIAL

Impairments in cognitive control using a reverse visually-guided reaching task following stroke

Catherine R. Lowrey Ph.D.[1], Sean P. Dukelow M.D. Ph.D.[2], Stephen D. Bagg M.D.[3,4], Ben Ritsma M.D.[3,4], Stephen H. Scott Ph.D.[1,3,4].

Affiliations

1. Centre for Neuroscience Studies, Queen's University, Kingston, ON, Canada
2. Hotchkiss Brain Institute, University of Calgary, Calgary, Alberta, Canada
3. Department of Physical Medicine and Rehabilitation, Queen's University, Kingston, Ontario, Canada
4. School of Medicine, Queen's University, Kingston, Ontario, Canada
5. Department of Biomedical and Molecular Sciences, Queen's University, Kingston, ON, Canada
6. Department of Medicine, Queen's University, Kingston, ON, Canada

Email:

Catherine Lowrey* – lowrey@queensu.ca

Sean Dukelow - sean.dukelow@albertahealthservices.ca

Stephen Bagg – baggs@providencecare.ca

Ben Ritsma - ritsmab@providencecare.ca

Stephen Scott - steve.scott@queensu.ca

Corresponding author: Catherine R. Lowrey, Laboratory of Integrative Motor Behaviour, Centre for Neuroscience Studies, 18 Stuart St., Queen's University, Kingston, ON, K7L 3N6, Canada, Phone: +1-613-533-6360, Fax: +1-613-533-6840, Email: lowrey@queensu.ca

Supplemental Methods

Full detail of the algorithms to determine different parts of the reaching movements:

i. Movement onset - determined algorithmically, first by finding the point in time when the hand cursor left and stayed out of the start target. Once this point was identified, movement onset was defined by moving back in time until either a local minimum in hand speed was found that was lower than the 95th percentile of posture speed or the hand speed dropped below the 50th percentile of posture speed (percentiles for posture speed were calculated using all reaches on that arm).
ii. Movement offset - determined by an algorithm that identified the time when the hand cursor entered the end target and moving forward in time until the hand speed dropped below the 95th percentile of posture speed or the hand speed drops below the 50th percentile of posture speed (percentiles for posture speed were calculated using all reaches on that arm).
iii. Initial movement – the initial phase of the reaching movement. This initial movement was determined as the time from movement onset to either the first speed minima or movement offset, whichever occurred first.
iv. False start – when a movement onset was detected at < 130 ms after the end target was turned on.

Full detail of the calculation of the Task Parameters (KST Standard Test Summary; https://kinarm.com/download/kst-summary-analysis-version-3-9/):

1. Posture Speed- Median hand speed when the hand should be at rest within a target. The median of all reaches was calculated.

2. Reaction Time – The time between the appearance of the target and movement onset. The median value of all reaches was calculated.

3. Initial Direction Angle – the angular deviation between a straight line from the hand cursor position at movement onset and the end of the initial movement, and a straight line from cursor position at movement onset and the end target. The median of all trials was calculated.

4. Initial Distance Ratio – the ratio of the hand path distance during the initial movement to the hand path distance for the whole movement (movement onset to movement offset). The median of all reaches was calculated.

5. Initial Speed Ratio – the ratio between the max speed during the initial movement to the max speed for the whole movement (movement onset to movement offset; i.e. Max Speed). The mean of all reaches was calculated.

6. Speed Maxima Count – the number of individual maxima found for the whole movement (movement onset to movement offset). The mean of all reaches was calculated.

7. Min-Max Speed – mean difference between sets of adjacent hand speed maxima and minima from Max Speed to movement onset. The mean of all reaches was calculated.

8. Movement Time – the total time for the whole movement (movement onset to movement offset). The median of all reaches was calculated.

9. Path Length Ratio – the ratio of the hand path distance for the whole movement (movement onset to movement offset) and the straight line distance between the hand position at movement onset and movement offset. The mean of all reaches was calculated

10. Max Speed – the maximum hand speed for the whole movement (movement onset to movement offset). The median of all reaches was calculated.

11. Direction Errors – The number of times the subjects initially moved the cursor in a direction away from the end target.

12. Correction Time – If a direction error occurs, the correction time is the amount of time before the subject started to move the cursor back towards the end target. If the initial movement of the cursor was toward the end target then the value for that trial is zero. Otherwise the correction time is calculated as the amount of time from the end target turning on to the time the subject was the farthest from the end target.

13. No Initial Stabilization – the number of reach trials where the subject failed to stabilize at the start target.

14. No Reaction Time – the number of reach trials where no movement onset could be calculated.

15. No End Movement – the number of reach trials where movement offset could not be detected before the end of the trial. Trials with false starts are not included in this count.

16. End Target Not Reached – the number of trials where the end target was not reached. Trials with false starts are not included in this count.

Reach trials were excluded from calculations if movement onset could not be detected, or a false start was detected. There were 5 ‘catch’ trials in which no peripheral target was presented.

Supplementary Tables

Table 1: Clinical test information

Clinical Test	Acronym	Description
Edinburgh Handedness Inventory	EHI	Used to assess the hand dominance and laterality (right or left) in everyday activities (e.g., writing, drawing, using scissors, opening a box, kicking a ball).
Montreal Cognitive Assessment	MoCA	Detects mild cognitive impairment and assesses attention, concentration, executive functions, memory, language, visuo-constructional skills, conceptual thinking, calculations and orientation. Scored out of 30, a score below 26 indicates mild cognitive impairment.[1]
Behavioural Inattention Test	BIT	Consists of 6 pencil and paper tests (e.g. line bisection, letter cancelation, copying, figure drawing) to screen for the presence of visual neglect. The test is scored out of 146 and a value less than 130 is indicative of visual neglect.[2]
Chedoke McMaster Stroke Assessment (arm and hand)	CMSA	The arm and hand sections of the CMSA each assess the upper limb on a 7-point scale reflecting stages of motor recovery (7-highest recovery stage, 1 - lowest recovery).[3]
Functional Independence Measure	FIM	Used to rate physical and cognitive disability and level of assistance required, intended to measure the burden of care. The motor portion (FIM motor) measures functional ability (e.g. washing, dressing, toileting) and mobility. The cognitive portion (FIM cognitive) evaluates comprehension, expression, social interaction, problem solving and memory.[4]

Table 2: Z-score cutoffs for parameters

**Parameter**	**Z cutoff**	**Incl. in Task score calc.**
Posture Speed	> 1.65	Y
Reaction Time	> 1.65	Y
Initial Direction Angle	> 1.65	Y
Initial Distance Ratio	< - 1.65	Y
Initial Speed Ratio*	< - 1.65*	Y*
Speed Maxima Count	> 1.65	Y
Min-Max Speed	> 1.65	Y
movement Time	> 1.65	Y
Path Length Ratio	> 1.65	Y
Max Speed	< - 1.65	Y
Direction Errors	> 1.65	Y
Correction Time	> 1.65	Y
No Initial Stabilization	> 1.65	Y
No Reaction Time	> 1.65	N
No end movement	> 1.65	Y
End target not reached	> 1.65	N
**Task Score**	**> 1.65**	**-**

*this parameter was unable to be transformed to normal for VGR only. Therefore the raw values were used to determine whether performance was outside 95% (< 0.95 speed ratio). This parameter did not contribute to the Task Score calculation for VGR

Table 3: Control participant information

	VGR	RVGR
Number of subjects	514	288
Age	18-93	18-84
Sex	220 M/ 294 F	127 M/161 F

Table 4: Results for VGR and RVGR: Percentage of participants with stroke with impairments in each parameter for each task

	VGR				RVGR			
**Parameter**	**Affected arm (% of participants impaired)**	**Total (#)†**	**Less Affected (%)**	**Total (#)**	**Affected arm (%)**	**Total (#)**	**Less Affected (%)**	**Total (#)**
Posture Speed	**34.7**	49	**20.1**	58	**41.5**	41	**32.8**	58
Reaction Time	**65.5**	55	**41.4**	58	**44.2**	52	**28.8**	59
Initial Direction Angle	**69.1**	55	**44.8**	58	**67.3**	52	**64.4**	59
Initial Distance Ratio	**78.2**	55	**31.1**	58	**5.8**	52	**3.7**	59
Initial Speed Ratio*	**47.3**	55	**17.24**	58	**42.3**	52	**32.2**	59
Speed Maxima Count	**58.2**	55	**10.3**	58	**73.1**	52	**52.5**	59
Min-Max Speed	**43.6**	55	**15.5**	58	**50**	52	**38.9**	59
movement Time	**60**	55	**13.8**	58	**78.8**	52	**50.9**	59
Path Length Ratio	**60**	55	**15.5**	58	**69.2**	52	**57.6**	59
Max Speed	**25.5**	55	**12.1**	58	**21.2**	52	**18.6**	59
Direction Errors	**-**	-	**-**	-	**3.44**	58	**20.3**	59
Correction Time	**-**	-	**-**	-	**44.2**	52	**54.2**	59
No Initial Stabilization	**49.2**	59	**16.9**	59	**71.2**	58	**42.4**	59
No Reaction Time	**52.5**	59	**16.9**	59	**71.2**	58	**40.7**	59
No end movement	**44.1**	59	**5.1**	59	**71.2**	58	**42.4**	59
End target not reached	**52.5**	59	**16.9**	59	**75.9**	58	**50.8**	59
**Task Score**	**81.3**	**59**	**50.8**	**59**	**86.2**	**58**	**72.9**	**59**

*parameter unable to be transformed to normal for VGR only. Raw values were used to determine whether performance was outside 95% (< 0.95 speed ratio). This parameter did not contribute to the Task Score calculation for VGR

† not enough trials existed for some participants to calculate a valid Z-score for some parameters so they were excluded from the overall count. Total number of participants used for each parameter (out of 59 who completed the task) is indicated here.

Table 5: Clinical Correlations

	VGR				RVGR			
	Affected		Less Affected		Affected		Less Affected	
**Clincial Test**	**rho**	**p_{adj}**	**rho**	**p_{adj}**	**rho**	**p_{adj}**	**rho**	**p_{adj}**
MoCA	-0.13	1.0**	**-0.39**	**0.02**	-0.093	1.0**	-0.23	0.78
FIM - motor	**-0.69**	**0.000***	-0.17	1.0**	**-0.44**	**0.02***	-0.042	1.0**
FIM - cog	-0.12	1.0**	-0.26	0.73	-0.19	1.0**	-0.14	1.0**
FIM - Total	**-0.64**	**0.000***	-0.18	1.0**	**-0.42**	**0.039***	-0.04	1.0**
CMSA arm	**-0.82**	**0.000***	-0.24	0.79	**-0.65**	**0.000***	-0.28	0.38
CMSA hand	**-0.76**	**0.000***	**-0.43**	**0.01**	**-0.57**	**0.000***	**-0.41**	**0.018***
BIT	-0.33	0.11	**-0.42**	**0.01**	-0.2	1.0**	-0.23	0.92

*P<0.05 ** P > 1.0 due to Bonferroni-adjustment of p-values, p is multiplied by the number of comparisons

Supplemental References

1. Nasreddine ZS, Phillips NA, Bédirian V, et al. The Montreal Cognitive Assessment, MoCA: a brief screening tool for mild cognitive impairment. *J Am Geriatr Soc*. 2005;53(4):695-699. doi:10.1111/j.1532-5415.2005.53221.x

2. Wilson B, Cockburn J, Halligan P. Development of a behavioral test of visuospatial neglect. *Arch Phys Med Rehabil*. 1987;68(2):98-102.

3. Gowland C, Stratford P, Ward M, et al. Measuring physical impairment and disability with the Chedoke-McMaster Stroke Assessment. *Stroke*. 1993;24(1):58-63.

4. Granger CV, Cotter AC, Hamilton BB, Fiedler RC. Functional assessment scales: a study of persons after stroke. *Arch Phys Med Rehabil*. 1993;74(2):133-138.
